# Evaluation of the Molecular Structural Parameters of Normal Rice Starch and Their Relationships with Its Thermal and Digestion Properties

**DOI:** 10.3390/molecules22091526

**Published:** 2017-09-12

**Authors:** Lingshang Lin, Qing Zhang, Long Zhang, Cunxu Wei

**Affiliations:** 1 Key Laboratory of Crop Genetics and Physiology of Jiangsu Province/Key Laboratory of Plant Functional Genomics of the Ministry of Education, Yangzhou University, Yangzhou 225009, China; 18252713442@163.com (L.L.); 18705275281@163.com (Q.Z.); zhanglong@yzu.edu.cn (L.Z.); 2 Co-Innovation Center for Modern Production Technology of Grain Crops of Jiangsu Province/Joint International Research Laboratory of Agriculture & Agri-Product Safety of the Ministry of Education, Yangzhou University, Yangzhou 225009, China

**Keywords:** starch, molecular structural parameters, thermal properties, digestion properties, correlation analysis

## Abstract

The molecular structural parameters of six normal rice starches with different amylose contents were investigated through their iodine absorption spectra and gel permeation chromatography of fully branched and debranched starches. The thermal and digestion properties of starches were also determined and their relationships with molecular structural parameters were analyzed. Results showed that the molecular structural parameters of maximum absorption wavelength, blue value (BV), optical density 620 nm/550 nm (OD 620/550), amylose, intermediate component, and amylopectin, including its short branch-chains, long branch-chains, and branching degree, had high correlation in different determining methods. The intermediate component of starch was significantly positively related to amylose and negatively related to amylopectin, and the amylopectin branching degree was significantly positively related to amylopectin content and negatively related to amylose content. The gelatinization temperatures and enthalpy of native starch were significantly positively related to BV, OD 620/550, and amylose content and negatively related to amylopectin short branch-chains. The gelatinization temperatures and enthalpy of retrograded starch were significantly negatively related to amylopectin branching degree. The digestions of gelatinized and retrograded starches were significantly negatively related to the BV, OD 620/550, amylose, and intermediate component and positively related to amylopectin and its short branch-chains and branching degree.

## 1. Introduction

Starch contains two main glucan polymers in its molecular structure: highly branched amylopectin and mainly linear amylose [1]. The molecular structure has significant effects on the thermal and digestion properties of starch, which are the two important functional properties that determine its applications in the food and non-food industries [2,3].

The molecular structure of starch includes the content and molecular weight distribution of amylose and amylopectin and is normally analyzed using the corresponding iodine absorption spectra and gel permeation chromatography (GPC) of fully branched and debranched starch [1,2,3]. Amylose and amylopectin have different iodine affinity values, producing starches that exhibit different iodine absorption spectra according to the different contents and molecular weight distributions of amylose and amylopectin [1,4]. Normally, the starch-iodine absorption spectrum is converted to maximum absorption wavelength (λ_max_), blue value (BV, absorbance at 680 nm), optical density 620 nm/550 nm ratio (OD 620/550), and apparent amylose content (AAC) [1,4,5]. Amylose can complex the lipid in starch, and the long branch-chains of amylopectin have iodine affinity properties similar to amylose. Therefore, AAC frequently underestimates or overestimates the amylose content (AC) of starch [6]. GPC can detect the molecular weight distribution of starch. For fully branched starch, GPC can separate starch into two fractions, amylopectin and amylose, or three fractions, amylopectin, intermediate component, and amylose [7]. For debranched starch, GPC can separate starch into three fractions, amylopectin short branch-chains, amylopectin long branch-chains, and amylose [8]. Though the molecular structures of starches have been reported in many papers, the molecular structural parameters derived from different analytical methods, which reflect different structural information, are seldom compared in previous works.

The thermal properties of starch are important indicators of starch quality. The morphology, size, molecular structure, and crystalline structure of starch all have significant effects on its thermal properties [9,10,11]. Starch contains three components: rapidly digestible starch (RDS), slowly digestible starch (SDS), and resistant starch (RS), according to their hydrolysis rates. The digestion properties of native starches are influenced by their morphologies, sizes, components, and crystalline structures but those of gelatinized and retrograded starches are affected mainly by their molecular structures [11,12,13,14]. For the starch applications, interrelationship between molecular structure and thermal and digestion properties must be understood. However, few papers report the relationships between thermal and digestion properties and molecular structural parameters derived from different analytical methods in one paper.

In this study, we determined and compared many molecular structural parameters of six normal rice starches using their iodine absorption spectra and GPC. The thermal and digestion properties of starches were also investigated and their relationships with molecular structural parameters were analyzed. Our objective is to evaluate the molecular structural parameters of starch and their relationships with functional properties and provide important information for starch application through determining the molecular structural parameters.

## 2. Results and Discussion

### 2.1. Molecular Structural Parameters of Starch

**Figure 1:**
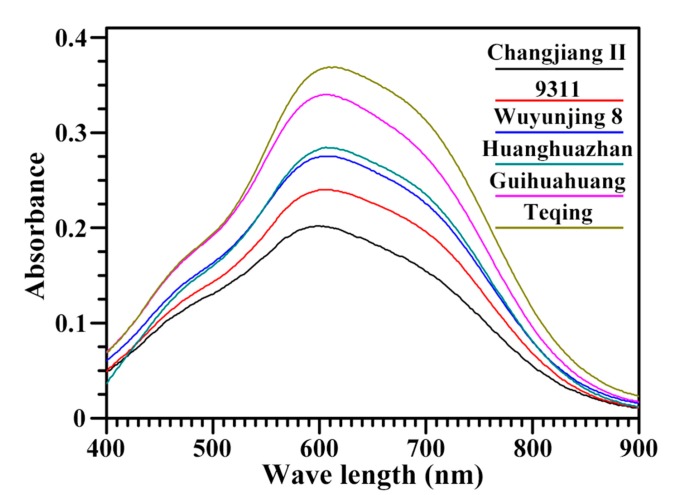
Iodine absorbance spectra of starches.

The absorption spectra of starch–iodine complexes were clearly different among the six rice starches (Figure 1). λ_max_ is related to the polymerization degree and average chain length of amylose and amylopectin. BV reflects the iodine affinity of starch [1,4]. OD 620/550 can indicate the relative content of longer chain segments in starch [5]. AAC reflects the iodine absorbance from both amylose and small amount of amylopectin with longer branch-chains and is commonly assessed from the absorbance value at 620 nm [15]. The λ_max_, BV, OD 620/550 nm, and AAC of starches are presented in Table 1 and ranged from 599.2 nm to 612.3 nm, 0.166 to 0.334, 1.150 to 1.305, and 10.4% to 27.9% in six rice varieties with the lowest value in Changjiang II and the highest value in Teqing.

**Figure 2:**
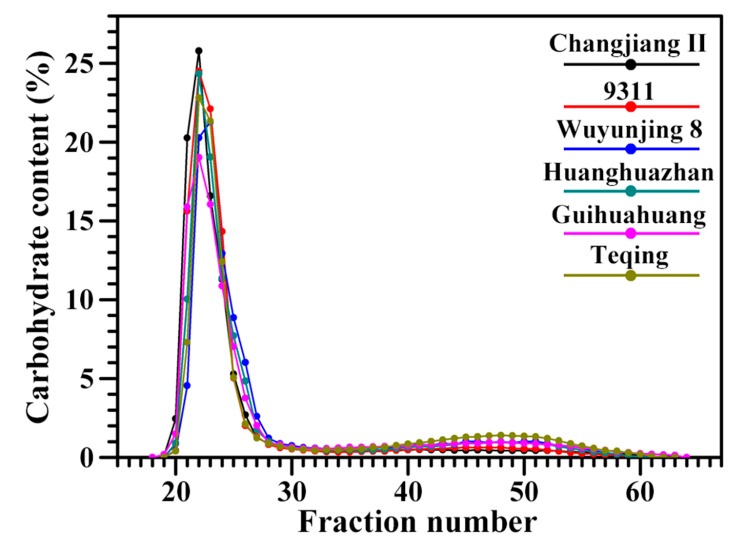
GPC profiles of fully branched starches.

The molecular weight distributions of fully branched starches are shown in Figure 2. Two starch fractions, amylopectin and amylose, are generally detected in the Sepharose CL-2B GPC profile of fully branched starch [7,14]. Several studies also divide the Sepharose CL-2B GPC profile of fully branched starch into three fractions: high, intermediate, and low molecular fraction. The high and low molecular fractions are mostly amylopectin and amylose, respectively, and the intermediate molecular fraction (intermediate component) is between amylopectin and amylose [16,17]. In this study, the fully branched starch was divided into three fractions according to elution times. The starches in the fractions 17–25, 26–39, and 40–60 were the high, intermediate, and low molecular fragments, respectively, and their contents were significantly different among the six rice starches (Table 2). Changjiang II starch had the highest amylopectin and lowest intermediate component and amylose content, and Teqing starch had the lowest amylopectin content and highest intermediate component and amylose content.

**Figure 3:**
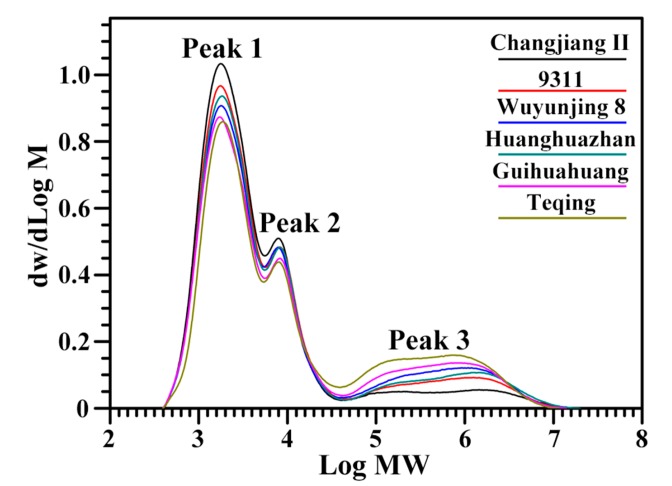
GPC chromatograms of isoamylase-debranched starch.

The GPC chromatograms of debranched starches are shown in Figure 3 and showed three clear peaks. Peak 1 consists of amylopectin short branch-chains (A and short B chains), peak 2 contains amylopectin long branch-chains (long B chains), and peak 3 is amylose [8]. The branching degree of amylopectin can be reflected by the area ratio of peak 1 to peak 2; the ratio increased with increasing branching degree [18]. The contents of amylopectin short branch-chains, amylopectin long branch-chains, and amylose and the area ratio of peak 1 to peak 2 in six rice starches are summarized in Table 2 and ranged from 53.8% to 68.7%, 20.2% to 21.1%, 10.2% to 26.0%, and 2.66% to 3.25%, respectively.

### 2.2. Correlation among Different Molecular Structural Parameters of Starch

The correlations among different molecular structural parameters are shown in Table 3. The λ_max_, BV, OD 620/550, and AAC derived from starch–iodine absorbance spectrum had highly positive correlation (*p* < 0.01), and were all positively related to amylose (AC_B_ and AC_D_) (*p* < 0.05) and negatively related to amylopectin (AP, AP-S, AP-L) and branching degree (AP-S/L) (*p* < 0.05). For molecular weight distribution of fully branched starch determined by sepharose CL-2B GPC, amylopectin content had significantly negative correlation with the contents of intermediate component and amylose (*p* < 0.01), and intermediate component showed high correlation with AC_B_ (*p* < 0.01). Similar correlation was also reported in previous studies [19,20]. For molecular weight distribution of debranched starch, the short branch-chains, long branch-chains, and branching degree of amylopectin had positive correlation (*p* < 0.05) and were all negatively related to amylose (AC_D_) (*p* < 0.05). The results are in agreement with previous reports in rice [21,22]. Both intermediate component and AC_B_ derived from Sepharose CL-2B GPC were positively related to amylose (AC_D_) derived from debranched starch (*p* < 0.05) but negatively related to the short branch-chain, long branch-chain, and branching degree of amylopectin (*p* < 0.05). Similar relationship is also reported in maize starch [3]. The above mentioned results indicated that the different molecular structural parameters determined by different methods had high correlation and reflected different structural information of starch.

### 2.3. Thermal Properties of Native and Retrograded Starches

**Figure 4:**
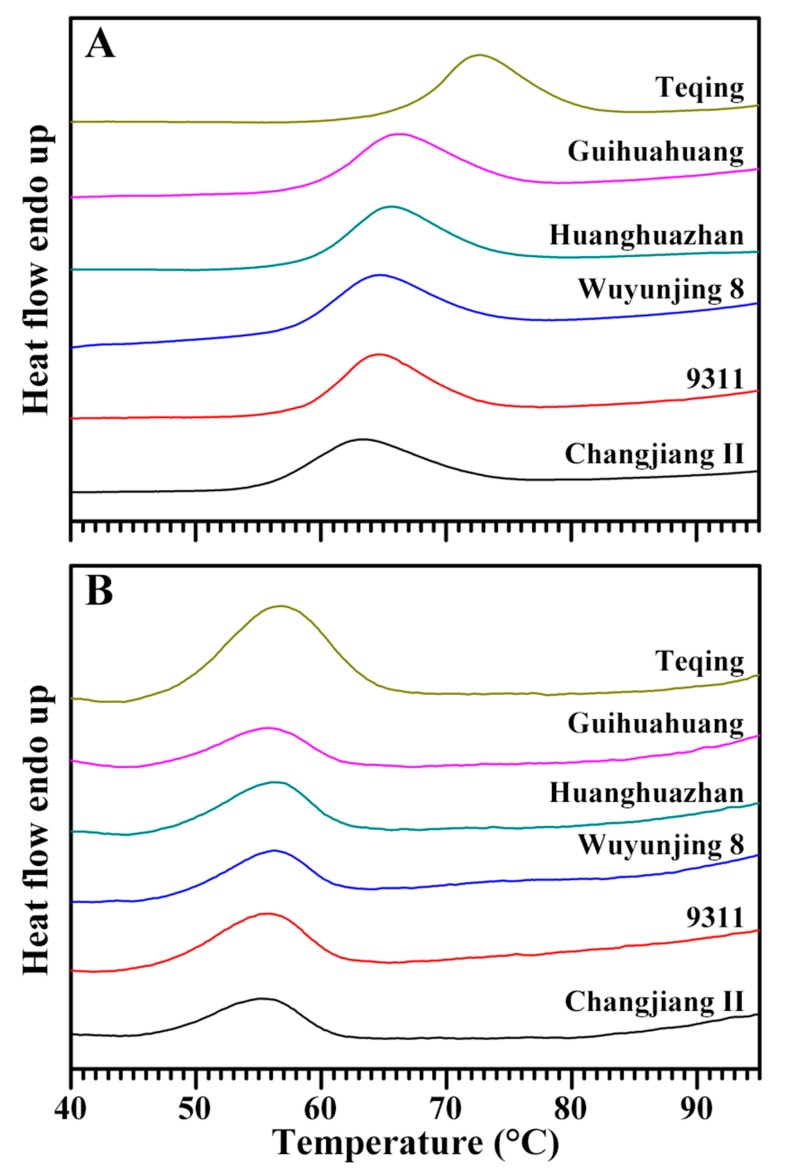
DSC thermograms of native starches (**A**) and retrograded starches (**B**).

The DSC thermograms of native and retrograded starches are presented in Figure 4 and their thermal parameters are listed in Table 4. The native starches of six rice varieties displayed different gelatinization temperatures but similar enthalpy. After storage at 4 °C for seven days, the gelatinization temperatures of retrograded starch were lower than those of native starch, indicating that the crystallites in retrograded starch were gelatinized more easily than those in native starch. The gelatinization temperatures of retrograded starches did not vary considerably among six rice starches, suggesting similarities in the melting behavior of crystallites in retrograded starch, but gelatinization enthalpy and retro% had significant difference, which is consistent with previous results [23,24].

### 2.4. Correlation between Molecular Structural Parameters and Thermal Properties of Starch

The correlations between molecular structural parameters and thermal parameters of rice starches are presented in Table 5. The gelatinization temperatures of native starches and the peak temperatures of retrograded starches were significantly positively related to BV and AC (*p* < 0.05), but negatively related to amylopectin short branch-chains and branching degree (*p* < 0.05). These data partly contradicted the previous studies. Park et al. found that gelatinization temperature correlated positively with AC and amylopectin long branch-chain, but no significant correlation was observed between gelatinization properties and amylopectin short branch-chains [10]. However, further studies showed that gelatinization temperature was significantly positively related to AC and negatively related to amylopectin short branch-chains [11,25,26]. In the present study, the gelatinization enthalpy of native starch was significantly positively related to λ_max_, BV, OD 620/550, and AC (*p* < 0.05) but negatively related to intermediate component and amylopectin, including short and long branch-chains (*p* < 0.05). However, the enthalpy and retro% of retrograded starch were only significantly positively related to amylose (AC_D_) and negatively related to amylopectin short branch-chains and branching degree (*p* < 0.05) (Table 5). Shi and Seib reported that amylopectin short branch-chains inhibited the retrogradation of starch [27].

### 2.5. In Vitro Digestion Properties of Gelatinized and Retrograded Starches

In vitro digestion properties of gelatinized and retrograded starches by both α-amylase from porcine pancreatic (PPA) and amyloglucosidase from *Aspergillus niger* (AAG) are shown in Table 6. The RDS, SDS, and RS in six rice varieties ranged from 73.6% to 83.7%, 8.9% to 9.7%, and 7.3% to 15.0% in gelatinized starches and 68.7% to 76.7%, 7.5% to 10.1%, and 15.2% to 22.8% in retrograded starches, respectively. The retrogradation of gelatinized starch can significantly increase the resistance to in vitro digestion [21,28].

### 2.6. Correlation between Molecular Structural Parameters and Digestion Properties of Starch

The correlation between molecular structural parameters and in vitro digestion properties of rice starches are presented in Table 7. The RDS of gelatinized and retrograded starches was significantly negatively related to BV, OD 620/550, AC, and intermediate component (*p* < 0.05) but positively related to amylopectin and its short branch-chains and branching degree (*p* < 0.01). The RS of gelatinized starches was significantly positively related to BV, OD 620/550, AC, and intermediate component (*p* < 0.05) but negatively related to amylopectin, including its short branch-chains, long branch-chains, and branching degree (*p* < 0.01). However, the RS of retrograded starches was only positively related to OD 680 and AC (*p* < 0.05) and negatively related to amylopectin short branch-chains and branching degree (*p* < 0.05). Several studies also showed that the digestibility of starch is significantly negatively related to AC but positively related to amylopectin short branch-chains [11,13,14,21]. For gelatinized starch, the leaching amylose complexes with lipids upon heating and reduces the enzyme susceptibility. Amylopectin short branch-chains are remarkably short for complex formation [29]. For retrograded starch, amylose that possess a more linear and flexible structure than amylopectin can form double helices (retrogradation) in a short time after gelatinization and has higher resistance toward amylase hydrolysis than amorphous starch [30]. Therefore, retrograded starch that contains high AC was digested more slowly than that with high content of amylopectin short branch-chains.

## 3. Materials and Methods

### 3.1. Plant Materials

Six normal rice varieties of 9311, Changjiang II, Guihuahuang, Huanghuazhan, Teqing, and Wuyunjing 8 were selected as plant materials in this study. The varieties of Changjiang II, Guihuahuang, and Wuyunjing 8 are *Japonica* rice and the others are *Indica* rice. They were grown in the experiment field of Yangzhou University (Yangzhou, China) in May 2015 and harvested in September 2015.

### 3.2. Isolation of Starch

Starch was isolated from mature brown seeds as previously described [31].

### 3.3. Measurements of Iodine Absorption Spectrum and AAC of Starch

The protein was removed from starch granules by using protease and sodium bisulfite. Starch was defatted with 85% (*v*/*v*) methanol and dissolved in urea dimethyl sulfoxide solution as previously described [32]. The iodine absorption spectrum of starch was determined using an Ultrospec 6300 pro spectrophotometer (Amersham Bioscience, Cambridge, UK), and AAC was measured from the absorbance at 620 nm as previously described [4].

### 3.4. Molecular Weight Distribution of Fully Branched Starch

The fully branched starch was analyzed as previously described [19] with some modifications. Eighteen milligrams of starch was treated using protease and sodium bisulfite to remove the protein from granules and dispersed in 1.8 mL DMSO at 80 °C overnight using a ThermoMixer (Eppendorf, Hamburg, Germany) at 350 rpm. Afterward, the sample was centrifuged (10 min, 4000× *g*). The supernatant (1 mL) was mixed with 4 mL of absolute ethanol to precipitate the starch, and the residual supernatant was used for subsequent debranched starch in Section 3.5. The precipitated starch was washed twice with ethanol and dispersed in warm deionized water (5 mL) and incubated for 30 min in boiling water. After cooling to room temperature, the sample was centrifuged (10 min, 4000× *g*) and the supernatant was sieved through a 5 μm filter. Two milliliters of supernatant was injected into a GPC column (1.6 cm × 50 cm) packed with Sepharose CL-2B (CL2B300 Sigma-Aldrich, St. Louis, MO, USA). The column was eluted at 0.5 mL/min with 25 mM NaCl and 1 mM NaOH solution, and 1.5 mL fractions were collected. The quantity of carbohydrate was determined using anthrone-H_2_SO_4_ method.

### 3.5. Molecular Weight Distribution of Debranched Starch

The starch in the residual supernatant in Section 3.4 was debranched with isoamylase as previously described [33]. The debranched starch was analyzed by a high-temperature GPC system equipped with three columns (PL110-6100, 6300, 6525) and a differential refractive index detector (PL-GPC 220, Agilent Technologies UK Limited, Shropshire, UK) as previously described [28].

### 3.6. Thermal Property Analysis of Starch

The thermal properties of native starch were measured by a differential scanning calorimeter (DSC200-F3, NETZSCH, Selb, Germany) as previously described [28]. Five milligrams of starch and fifteen microliters of distilled water were mixed and sealed in an aluminum pan at 4 °C overnight. The sample was equilibrated at room temperature for 1 h and then heated to 130 °C at 10 °C/min. The gelatinized starch was retrograded at 4 °C for seven days and rescanned at the same condition as native starch.

### 3.7. In Vitro Digestion Analysis of Gelatinized and Retrograded Starch

The in vitro digestion of starch was determined using both PPA (A3176, Sigma) and AAG (E-AMGDF, Megazyme, Bray, Ireland) as previously described [34]. Briefly, native starch (10 mg) was gelatinized in a 10 mL centrifuge tube with 1 mL distilled water using ThermoMixer at 1000 rpm for 12 min at 98 °C. The gelatinized starch was recrystallized at 4 °C for 36 h for preparing the retrograded starch. The gelatinized or retrograded starch was equilibrated at 37 °C for 30 min, and 1 mL of enzyme solution (20 mM sodium phosphate buffer, pH 6.0, 6.7 mM NaCl, 0.01% NaN_3_, 2.5 mM CaCl_2_, 4 U PPA, 4 U AAG) was added in the centrifuge tube. The sample was incubated at 37 °C for 20 or 120 min using ThermoMixer at 1000 rpm, and then 240 μL of 0.1 M HCl and 2 mL of 50% ethanol were added immediately to terminate the hydrolysis. The sample was centrifuged (14,000× *g*, 5 min), and the glucose quantity in the supernatant was determined using the glucose assay kit (Megazyme, K-GLUC). The digested starches within 20 min and between 20 to 120 min were RDS and SDS, respectively, and the undigested starch at 120 min was RS.

### 3.8. Statistical Analysis

Analysis of variance of the means, comparisons of multiple means using Tukey’s multiple-range tests at *p* < 0.05, and Pearson’s bivariate correlations were performed using SPSS 19.0 software (IBM Company, Chicago, IL, USA).

## 4. Conclusions

The molecular structural parameters of starch measured by different methods could reflect different structural information and had significant correlation. The intermediate component of starch was significantly positively related to amylose and negatively related to amylopectin including its short branch-chains, long branch-chains, and branching degree. The gelatinization temperatures of native starches and peak temperature of retrograded starches were significantly positively related to BV, OD 620/550, and AC and negatively related to amylopectin, including its short branch-chains and branching degree. The gelatinization enthalpy and retro% of retrograded starch were significantly negatively related to amylopectin short branch-chains and branching degree and positively related to AC of debranched starches. The resistance of starch to in vitro digestion could be increased by the BV, OD 620/550, AC, and intermediate component and decreased by amylopectin, including its short branch-chains and branching degree.

## Figures and Tables

**Table 1:** Iodine absorbance spectrum parameters of starches ^a^.

Rice Variety	λ_max_ (nm)	BV ^b^	OD 620/550	AAC (%) ^b^
Changjiang II	599.2 ± 0.8 a	0.166 ± 0.004 a	1.150 ± 0.007 a	10.4 ± 0.4 a
9311	605.8 ± 0.6 b	0.211 ± 0.001 b	1.219 ± 0.001 b	15.5 ± 0.4 b
Wuyunjing 8	607.8 ± 0.6 bc	0.243 ± 0.001 c	1.225 ± 0.013 bc	18.4 ± 0.2 c
Huanghuazhan	609.8 ± 1.0 cd	0.255 ± 0.004 d	1.264 ± 0.019 cd	19.6 ± 0.4 d
Guihuahuang	610.2 ± 2.8 cd	0.300 ± 0.004 e	1.273 ± 0.033 d	24.7 ± 0.3 e
Teqing	612.3 ± 0.6 d	0.334 ± 0.002 f	1.305 ± 0.015 d	27.9 ± 0.4 f

^a^ Data are the means ± standard deviations, *n* = 3. Values in the same column with different letters are significantly different (*p* < 0.05). ^b^ BV, blue value; AAC, apparent amylose content.

**Table 2:** Molecular weight distributions of fully branched and debranched starches ^a^.

Rice Variety	Fully Branched Starch			Debranched Starch			
	AP (%) ^b^	IC (%) ^b^	AC_B_ (%) ^b^	AP-S (%) ^c^	AP-L (%) ^c^	AC_D_ (%) ^c^	AP-S/L ^c^
Changjiang II	87.1 ± 0.7 d	6.6 ± 0.6 a	6.3 ± 0.3 a	68.7 ± 0.2 d	21.1 ± 0.2 a	10.2 ± 0.1 a	3.25 ± 0.04 c
9311	85.2 ± 1.2 d	6.7 ± 0.6 a	8.0 ± 1.0 a	63.6 ± 0.9 c	20.8 ± 0.4 a	15.6 ± 1.3 b	3.05 ± 0.01 bc
Wuyunjing 8	79.0 ± 0.7 b	8.6 ± 0.6 bc	12.4 ± 0.4 bc	61.1 ± 1.2 c	20.5 ± 0.6 a	18.4 ± 0.6 c	2.98 ± 0.15 bc
Huanghuazhan	81.8 ± 0.7 c	7.5 ± 0.6 ab	10.7 ± 0.8 b	61.9 ± 0.7 c	20.7 ± 0.4 a	17.5 ± 1.1 bc	3.00 ± 0.02 bc
Guihuahuang	76.9 ± 0.9 b	8.8 ± 1.0 bc	14.3 ± 1.9 cd	58.7 ± 0.7 b	20.1 ± 0.4 a	21.2 ± 0.3 d	2.92 ± 0.09 b
Teqing	74.3 ± 1.2 a	9.2 ± 0.4 c	16.4 ± 1.2 d	53.8 ± 0.4 a	20.2 ± 0.2 a	26.0 ± 0.2 e	2.66 ± 0.05 a

^a^ Data are the means ± standard deviations, *n* = 3. Values in the same column with different letters are significantly different (*p* < 0.05). ^b^ AP, IC, and AC_B_ are the content of amylopectin, intermediate component, and amylose of fully branched starch, respectively. ^c^ AP-S, AP-L, and AC_D_ are the content of amylopectin short branch-chains, amylopectin long branch-chains, and amylose of debranched starch, respectively. AP-S/L is the content ratio of amylopectin short to long branch-chains.

**Table 3:** Pearson correlation coefficients between molecular structural parameters of starches ^a^.

	λ_max_	BV	OD 620/550	AAC	AP	IC	AC_B_	AP-S	AP-L	AC_D_
BV	0.936 **									
OD 620/550	0.983 **	0.962 **								
AAC	0.936 **	0.999 **	0.963 **							
AP	‒0.873 *	‒0.960 **	‒0.870 *	‒0.956 **						
IC	0.798	0.899 *	0.778	0.893 *	‒0.985 **					
AC_B_	0.890 *	0.973 **	0.892 *	0.969 **	‒0.999 **	0.976 **				
AP-S	−0.878 *	−0.945 **	−0.876 *	−0.949 **	0.947 **	−0.919 **	−0.965 **			
AP-L	−0.929 **	−0.979 **	−0.945 **	−0.979 **	0.954 **	−0.897 *	−0.949 **	0.904 *		
AC_D_	0.931 **	0.983 **	0.946 **	0.983 **	−0.959 **	0.904 *	0.970 **	−1.000 **	−0.917 *	
AP-S/L	−0.906 *	−0.955 **	−0.929 **	−0.953 **	0.921 **	−0.858 *	−0.934 **	0.992 **	0.842 *	−0.987 **

^a^ The abbreviations are shown in Table 1 and Table 2. * and ** indicate the significance at the *p* < 0.05 and *p* < 0.01 levels, respectively.

**Table 4:** Thermal parameters of native and retrograded starches ^a^.

Rice Variety	Native Starch					Retrograded Starch					
	T_o_ (°C) ^b^	T_p_ (°C) ^b^	T_c_ (°C) ^b^	ΔT (°C) ^b^	ΔH (J/g) ^b^	T_o_ (°C)	T_p_ (°C)	T_c_ (°C)	ΔT (°C)	ΔH (J/g)	Retro (%) ^b^
Changjiang II	56.1 ± 0.2 a	63.1 ± 0.4 a	72.5 ± 0.6 a	16.5 ± 0.8 d	9.88 ± 0.05 a	47.2 ± 1.6 a	54.8 ± 1.2 a	60.2 ± 1.3 a	13.0 ± 0.3 a	1.86 ± 0.02 a	18.8 ± 0.1 a
9311	58.4 ± 0.6 bc	64.7 ± 0.1 b	72.3 ± 0.4 a	13.9 ± 0.2 ab	10.01 ± 0.08 a	47.3 ± 3.1 a	55.2 ± 1.1 a	61.0 ± 0.5 a	13.7 ± 2.6 a	2.74 ± 0.30 ab	27.4 ± 3.2 b
Wuyunjing 8	58.1 ± 0.1 b	64.8 ± 0.1 b	73.2 ± 0.4 ab	15.1 ± 0.4 bcd	10.19 ± 0.47 a	47.2 ± 1.5 a	55.4 ± 1.2 a	60.7 ± 0.4 a	13.5 ± 1.1 a	2.03 ± 0.32 a	19.9 ± 2.2 a
Huanghuazhan	59.2 ± 0.1 cd	65.7 ± 0.1 c	74.1 ± 0.2 b	14.9 ± 0.3 bc	10.29 ± 0.43 a	47.3 ± 1.7 a	55.8 ± 0.8 a	61.5 ± 0.2 a	14.2 ± 1.5 a	3.06 ± 0.03 b	29.8 ± 1.6 b
Guihuahuang	59.7 ± 0.1 d	66.3 ± 0.1 d	75.2 ± 0.1 c	15.5 ± 0.1 cd	10.48 ± 0.09 a	47.3 ± 1.1 a	55.5 ± 1.0 a	61.3 ± 0.1 a	14.0 ± 1.1 a	3.13 ± 0.14 b	29.8 ± 1.1 b
Teqing	66.8 ± 0.2 e	72.2 ± 0.1 e	79.4 ± 0.1 d	12.7 ± 0.2 a	10.36 ± 0.13 a	48.3 ± 0.9 a	56.6 ± 0.4 a	64.2 ± 0.1 b	15.9 ± 1.0 a	6.41 ± 0.46 c	61.9 ± 3.6 c

^a^ Data are the means ± standard deviations, *n* = 3. Values in the same column with different letters are significantly different (*p* < 0.05). ^b^ T_o_, gelatinization onset temperature; T_p_, gelatinization peak temperature; T_c_, gelatinization conclusion temperature; ΔT, gelatinization temperature range (T_c_–T_o_); ΔH, gelatinization enthalpy; Retro, the 100% ΔH of dissociation of retrograded starch/ΔH of starch gelatinization.

**Table 5:** Pearson correlation coefficients between molecular structural parameters and thermal properties of starches ^a^.

	λ_max_	BV	OD 620/550	AAC	AP	IC	AC_B_	AP-S	AP-L	AC_D_	AP-S/L
N-T_o_	0.757	0.860 *	0.828 *	0.857 *	‒0.796	0.716	0.815 *	−0.906 *	−0.674	0.896 *	‒0.946 **
N-T_p_	0.757	0.873 *	0.831 *	0.869 *	−0.814 *	0.738	0.832 *	−0.911 *	−0.693	0.901 *	‒0.947 **
N-T_c_	0.717	0.880 *	0.800	0.873 *	−0.842 *	0.780	0.855 *	−0.885 *	−0.720	0.879 *	−0.908 *
N-ΔT	−0.659	−0.619	−0.691	−0.623	0.518	−0.421	−0.543	0.737	0.424	−0.720	0.801
N-ΔH	0.904 *	0.921 **	0.902 *	0.921 **	−0.883 *	−0.834 *	0.893 *	−0.840 *	−0.950 **	0.853 *	−0.775
R-T_o_	0.557	0.706	0.658	0.701	−0.637	0.558	0.656	−0.764	−0.481	0.749	−0.827 *
R-T_p_	0.876 *	0.901 *	0.917 *	0.893 *	−0.827 *	0.740	0.849 *	−0.926 **	−0.713	0.916 *	−0.955 **
R-T_c_	0.722	0.820 *	0.804	0.815 *	−0.738	0.648	0.760	−0.863 *	−0.606	0.850 *	−0.912 *
R-ΔT	0.780	0.856 *	0.852 *	0.851 *	−0.769	0.675	0.792	−0.891 *	−0.649	0.880 *	−0.934 **
R-ΔH	0.689	0.807	0.784	0.804	−0.713	0.619	0.737	−0.845 *	−0.598	0.833 *	−0.893 *
Retro	0.675	0.792	0.770	0.789	−0.696	0.601	0.720	−0.833 *	−0.578	0.820 *	−0.884 *

^a^ The N-T_o_, T_p_, T_c_, ΔT and ΔH are the DSC parameters of native starch; the R-T_o_, T_p_, T_c_, ΔT and ΔH are the DSC parameters of retrograded starch, and the other abbreviations are shown in Table 1, Table 2 and Table 4. * and ** indicate the significance at the *p* < 0.05 and *p* < 0.01 levels, respectively.

**Table 6:** Digestion properties of gelatinized and retrograded starches ^a^.

Rice Variety	Gelatinized Starch			Retrograded Starch		
	RDS (%) ^b^	SDS (%) ^b^	RS (%) ^b^	RDS (%)	SDS (%)	RS (%)
Changjiang II	83.7 ± 1.0 d	8.9 ± 0.6 a	7.3 ± 0.9 a	76.7 ± 1.3 d	7.6 ± 1.0 a	15.7 ± 0.3 a
9311	81.1 ± 0.6 c	9.4 ± 0.1 a	9.5 ± 0.6 b	76.6 ± 0.6 d	7.5 ± 0.3 a	15.9 ± 0.8 a
Wuyunjing 8	78.9 ± 0.4 b	9.6 ± 0.3 a	11.5 ± 0.1 c	73.5 ± 1.0 c	10.1 ± 0.6 b	16.5 ± 0.7 a
Huanghuazhan	80.7 ± 0.9 bc	9.4 ± 0.3 a	9.9 ± 0.6 b	74.7 ± 0.3 c	10.1 ± 0.6 b	15.2 ± 0.8 a
Guihuahuang	78.9 ± 0.6 b	9.7 ± 0.5 a	11.4 ± 0.8 c	71.6 ± 0.6 b	10.0 ± 0.3 b	18.5 ± 0.5 b
Teqing	73.6 ± 0.9 a	11.4 ± 0.4 b	15.0 ± 0.4 d	68.7 ± 0.3 a	8.4 ± 0.2 a	22.8 ± 0.4 c

^a^ Data are the means ± standard deviations, *n* = 3. Values in the same column with different letters are significantly different (*p* < 0.05). ^b^ RDS, rapidly digestible starch; SDS, slowly digestible starch; RS, resistant starch.

**Table 7:** Pearson correlation coefficients between molecular structural parameters and in vitro digestion properties of starches ^a^.

	G-RDS	G-SDS	G-RS	R-RDS	R-SDS	R-RS
λ_max_	−0.830 *	0.723	0.853 *	−0.799	0.575	0.603
BV	−0.914 *	0.849 *	0.922 **	−0.947 **	0.457	0.813 *
OD 620/550	−0.851 *	0.784	0.862 *	−0.834 *	0.472	0.686
AAC	−0.910 *	0.845 *	0.919 **	−0.942 **	0.446	0.812 *
AP	0.920 **	−0.825 *	−0.938 **	0.972 **	−0.526	−0.810
IC	−0.880 *	0.769	0.904 *	−0.948 **	0.552	0.772
AC_B_	−0.927 **	0.837 *	0.943 **	−0.973 **	0.515	0.816 *
AP-S	0.970 **	−0.908 *	−0.976 **	0.943 **	−0.365	−0.849 *
AP-L	0.806	−0.686	−0.834 *	0.888 *	−0.564	−0.703
AC_D_	−0.964 **	0.898 *	0.972 **	−0.945 **	0.381	0.844 *
AP-S/L	0.984 **	−0.945 **	−0.983 **	0.928 **	−0.284	−0.869 *

^a^ The G-RDS, SDS and RS are the digestion properties of gelatinized starch, the R-RDS, SDS and RS are the digestion properties of retrograded starch, and the other abbreviations are shown in Table 1, Table 2 and Table 6. * and ** indicate the significance at the *p* < 0.05 and *p* < 0.01 levels, respectively.

## Supplementary Materials

Supplementary File 1
