# Supplementary material for: Evaluation of the Molecular Structural Parameters of Normal Rice Starch and Their Relationships with Its Thermal and Digestion Properties
Source: Molecules. 2017 Sep 12;22(9):1526. doi: 10.3390/molecules22091526 (PMC6151547; doi:10.3390/molecules22091526)
Supplement: Supplementary File 1 [file molecules-22-01526-s001.pdf]

Certificate of English Editing

To whom it may concern:

This memo certifies that one of our clients has contracted our academic editing service for the following file.

Order Number:
P-201708300559jsy
Word Count:
2831 words
Date of the review:
08/30/17 (MM/DD/YY)

The English review was conducted using a two-stage process, in which a junior editor first reviewed the file, and then a senior editor conducted a final and more thorough review. All of our editors are native English-speaking professionals.

Documents receiving this certification should be English-ready for publication; however, the author has the ability to accept or reject our suggestions and changes.

We would like to emphasize that our service targets grammar and language edits. We do not rewrite the documents from scratch. If you are dissatisfied with specific revisions, please contact service@essaystar.com.

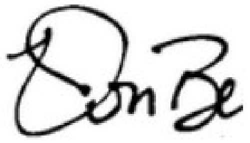

Essaystar Group

+1-208-975-4235
EssayStar, 93 S Jackson St, Seattle, WA 98104
